# Principal component analysis of biometric traits to reveal body confirmation in local hill cattle of Himalayan state of Himachal Pradesh, India

**DOI:** 10.14202/vetworld.2015.1453-1457

**Published:** 2015-12-29

**Authors:** Deepak Verma, Varun Sankhyan, Sanjeet Katoch, Yash Pal Thakur

**Affiliations:** Department of Animal Genetics and Breeding, College of Veterinary and Animal Sciences, Chaudhary Sarwan Kumar Himachal Pradesh Krishi Vishvavidyalaya, Palampur, Himachal Pradesh, India

**Keywords:** biometrical traits, hill cattle, phenotypic characterization, principal component analysis

## Abstract

**Aim:**

In the present study, biometric traits (body length [BL], heart girth [HG], paunch girth (PG), forelimb length (FLL), hind limb length (HLL), face length, forehead width, forehead length, height at hump, hump length (HL), hook to hook distance, pin to pin distance, tail length (TL), TL up to switch, horn length, horn circumference, and ear length were studied in 218 adult hill cattle of Himachal Pradesh for phenotypic characterization.

**Materials and Methods:**

Morphological and biometrical observations were recorded on 218 hill cattle randomly selected from different districts within the breeding tract. Multivariate statistics and principal component analysis are used to account for the maximum portion of variation present in the original set of variables with a minimum number of composite variables through Statistical software, SAS 9.2.

**Result:**

Five components were extracted which accounted for 65.9% of variance. The first component explained general body confirmation and explained 34.7% variation. It was represented by significant loading for BL, HG, PG, FLL, and HLL. Communality estimate ranged from 0.41 (HL) to 0.88 (TL). Second, third, fourth, and fifth component had a high loading for tail characteristics, horn characteristics, facial biometrics, and rear body, respectively.

**Conclusion:**

The result of component analysis of biometric traits suggested that indigenous hill cattle of Himachal Pradesh are small and compact size cattle with a medium hump, horizontally placed short ears, and a long tail. The study also revealed that factors extracted from the present investigation could be used in breeding programs with sufficient reduction in the number of biometric traits to be recorded to explain the body confirmation.

## Introduction

Biometric traits are used to characterize body confirmation of different breeds of livestock, compare growth in different animals and also describe an individual or population in a better way than the conventional methods of weighing and grading [1]. Characterization of the breed is the first approach to sustainable use of animal genetic resources. Phenotypic characterization is used to identify and document within and between breed variation of distinct breeds on their observable attributes [2].

Analysis of variance and correlation are widely used to characterize phenotypic and genetic relationship among body measurements of animals [3-5]. However, factor analysis using principal component analysis (PCA) is a valuable refinement statistical tool in multivariate methodology that is of use when characteristics are correlated [6]. The PCA transforms an original group of variables into another group, principal components, which are a linear combination of the original variables. In animal breeding, this technique is of use to simultaneously consider a group of attribute which may be used for selection and conservation purpose. Hill cattle of Himachal Pradesh belongs to indigenous Zebu breed of India known to possess gene combinations and special adaptation traits such as disease resistance, adaptation to harsh climate, and effective utilization of poor quality forages [7].

In spite of such unique characteristics hill cattle is among such cattle population of India which are not properly documented. Local cattle have their specific utilities in their native tract but in the last few decades, there is marked decline in their population due to widespread use of crossbreeding, destruction of the traditional production system and general thrust towards high input management system. To prevent the rich biological heritage from genetic erosion, it is imperative to redefine the breeding strategies and conservation programs. Biometric characterization is one of the important prerequisite along with other factors such as population size, geographical location, utility and management practices for implementation of breed improvement program. Furthermore, different biometric measurements, which represent the size of the animal is important criteria for selection of elite animals.

Therefore, the present investigation is planned with the objective of biometrical documentation of hill cattle by studying different body measurements and to develop latent factor to define which of these measures best represent body confirmation in local hill cattle of Himachal Pradesh.

## Materials and Methods

### Ethical approval

During biometric data collection, attention has been paid to minimize the discomfort to the animal and animal handling was carried out in accordance with guidelines laid down by the International Animal Ethics Committee and prevailing local laws and regulation.

### Study area and data collection for trait studied

**Figure-1:**
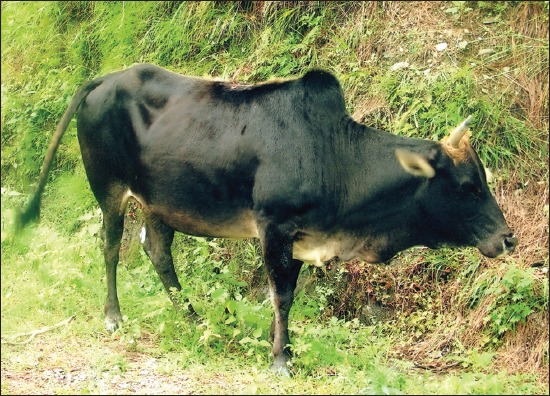
Adult hill cattle of Himachal Pradesh.

**Figure-2:**
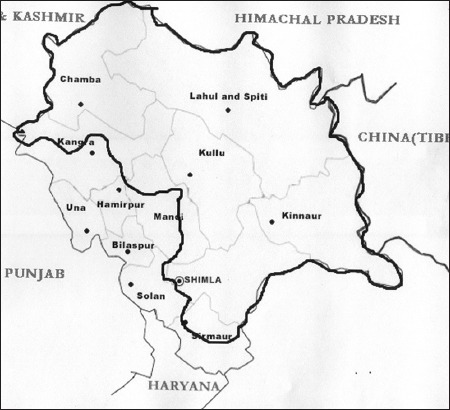
Map depicting the breeding tract of hill cattle of Himachal Pradesh.

The study was carried out in breeding tract of hill cattle (Figure-1) of Himachal Pradesh, India (Figure-2). The area lies in Himalayan region and is located at latitude 30°22’-34°12’ and longitude 75°47’-79°40’E, at altitude above 1800 m above mean sea level. Morphological and biometrical observations were recorded on 218 hill cattle (72 male and 146 females) randomly selected from different districts within the breeding tract which includes Shimla (42), Kinnaur (28), Kangra (48), Kullu (30) Chamba (28) and Mandi (40) districts. To avoid age and sex effect only adult hill cattle (3 years and above) were included in the study [1]. Proper care is exercised to avoid measuring crossbreds, unhealthy and pregnant animals. Due care was taken while recording to measure animal in upright position on the level surface. 17 morphometric traits were measured on each animal. Body measurements recorded include body length (BL), heart girth (HG), paunch girth (PG) forelimb length (FLL), hind limb length (HLL), face length (FL), forehead width (FOL), forehead length (FOW), height at hump (HH), hump length (HL), hook to hook distance (HHD), pin to pin distance (PPD), tail length (TL), TL up to switch (TUS), horn length (HOL), horn circumference (HC), and ear length (EL).

### Statistical analysis

Data collected were analyzed using fixed effect model, by considering district effect as fixed so as to adjust the data for significant effect of district if any as per the following statistical model [8].


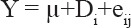


Where, Y is the phenotypic observation for one of the 17 biometric traits, µ is the overall mean; D_i_ is the fixed effect of district, while e_ij_ is the random residual error associated with each observation which is normally and independently distributed with mean zero and unit variance. Means, standard error and coefficient of variation were calculated. Pearson’s correlations (r) among various morphometric traits were estimated.

### PCA

The objective of PCA is to account for the maximum portion of variation present in the original set of variables with a minimum number of composite variables. Anti-image correlation, Kaiser–Meyer–Olkin (KMO) measure of sampling adequacy and Bartlett’s test of sphericity were computed to test the validity of the factor analysis of the data sets. The Eigen values were obtained by spectral decomposition of the data matrix and arrange in decreasing order of the corresponding eigenvalues, which equals to the variance of components. Thus, the first component has the largest variance. Varimax rotation was used for rotation of principal factors through the transformation of factors to approximate a simple structure. The estimate of communality for each variable measure the proportion of variance of that variable explained by all other factors jointly. All the analysis was performed using statistical software, SAS version 9.2.

## Results and Discussion

There was no significant effect of the district on all the traits studied in the hill cattle. Mean of biometric traits (cm) studied were 101.80±0.62 for BL, 137.38±1.08 for HG, 159.70±1.07 for PG, 26.70±0.11 for FLL, 38.57±0.21 for HLL, 39.31±0.22 for FL, 11.12±0.12 for FOL, 12.20±0.10 for FOW, 97.59±0.52 for HH, 3.24±0.07 for HL, 27.22±0.30 HHD, 12.43±0.08 for PPD, 68.04±0.55 for TL, 83.48±0.71 for TUS, 14.32±0.15 for HOL, 22.85±0.38 for HC 17.78±0.08 for EL. The morphometric characteristics observed in the present study suggested that indigenous hill cattle of Himachal Pradesh are small and compact size cattle with a medium hump, horizontally placed short ears and a long tail. The morphometric characteristics observed in the present study were more or less similar to other hill cattle of the country. Earlier study Pundir *et al*. [9] reported BL, HG and HH of hill cattle of neighboring state Uttarakhand as 98.75, 127.10 and 96.54 cm, respectively. In another study for Assam hill cattle of India [10] BL and HG were reported as 103.92 and 135.34 cm, respectively, similar to our finding but HH in hill cattle observed in the present investigation (97.59 cm) was comparatively lower than Assam Hill cattle (111.34 cm). Most of the biometric traits studied show less variability, indicating hill cattle are almost similar in confirmation. Coefficients of correlation among different traits are presented in Table-1. The study revealed that body measurements were mostly positively and significantly correlated (p<0.05). Correlation among confirmation traits ranged from −0.15 (EL and PPD) to 0.98 (HG and PG). A total of 136 correlation were estimated out of these 105 were significant of which all were positive except one (EL and PPD).

**Table-1:** Coefficient of phenotypic correlation among 17 biometric traits of hill cattle of Himachal Pradesh.

Trait	BL	CG	PG	FLL	HLL	FL	FOL	FOW	HH	HL	HHD	PPD	TL	TUS	HOL	HC	EL
BL		0.46**	0.49**	0.42**	0.42**	0.49**	0.27**	0.44**	0.64**	0.28**	0.47**	0.02	0.40**	0.48**	0.37**	0.27**	0.13
CG			0.80**	0.34**	0.39**	0.43**	0.13	0.27**	0.58**	0.25**	0.48**	0.03	0.27**	0.33**	0.30**	0.27**	0.04
PG				0.50**	0.35**	0.40**	0.11	0.23**	0.56**	0.26**	0.47**	0.03	0.31**	0.34**	0.31**	0.17**	0.06
FLL					0.53**	0.40**	0.19**	0.29**	0.39**	0.12	0.18**	0.02	0.13*	0.20**	0.23**	0.05	0.05
HLL						0.34**	0.22**	0.29**	0.41**	0.14*	0.26**	0.01	0.20**	0.27**	0.18**	0.19**	0.10
FL							0.79	0.39**	0.63**	0.18**	0.26**	−0.07	0.37**	0.42**	0.43**	0.31**	0.30**
FOL								0.59**	0.34**	0.26**	0.48**	−0.1	0.22**	0.24**	0.27**	0.16*	0.03
FOW									0.52**	0.31*	0.24**	−0.1	0.38**	0.40**	0.42**	0.30**	0.18*
HH										0.47**	0.02	0.05	0.51**	0.60**	0.55**	0.35**	0.21**
HL											0.03	0.08	0.16*	0.22**	0.21**	0.13*	0.02
HHD												0.27**	0.25**	0.26**	0.06	0.20**	−0.08
PPD													0.01	−0.1	−0.07	0.09	−0.15*
TL														0.82**	0.38**	0.22**	0.21**
TUS															0.38**	0.24**	0.17*
HOL																0.42**	0.28**
HC																	0.05
EL																	

Observation recorded were same for all traits.

** Significant at *P*<0.01;

* Significant at *P*<0.01. BL=Body length, HG=Heart girth, PG=Paunch girth, FLL=Forelimb length, HLL=Hind limb length, FL=Face length, FOL=Forehead length, FOW=Forehead width, HH=Height at hump, HL=Hump length, HHD=Hook to hook distance, PPD=Pin to pin distance, TL=Tail length, TUS=Tail length up to switch, HL=Horn length, HC=Horn circumference, EL=Ear length

**Figure-3:**
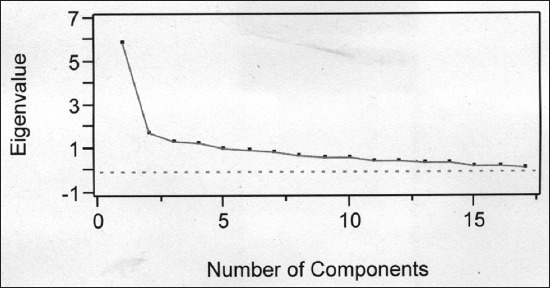
Scree plot showing component number with Eigen values.

Anti-image correlation analysis revealed that partial correlation was low, indicating that true factors existed in the data, which was further supported by KMO measure of sampling adequacy. The KMO observed in the present investigation was 0.75, comparatively higher than earlier reports on hill cattle [4] of Assam (0.60), whereas in study of other indigenous cattle, Kankrej [1] higher estimate (0.81) was reported. The KMO revealed the proportion of variance in different biometric traits caused by underlying factors. The overall significance of correlation tested with Bartlett’s test of sphericity for the biometric traits was significant and provided enough support for the validity of factor analysis of data. The estimated factor loading extracted by factor analysis, Eigen values and variation explained by each factor are presented in Table-2. There were five component extracted using Kaiser Rule criterion [11] to determine the number of component, i.e., retaining only the component that have eigenvalue >1 (Table-2). Another criterion for determination of number of component is scree plot that could be used to decide the actual number of component to be retained for analysis. Scree plot can depict various components and the component having eigenvalue up to the bent of elbow are usually considered (Figure-3). Extracted five principal components accounted for 65.9% of the variance in data. The first component was sufficient to explain about 34.7% of the total variance among 16 body measured estimated. The first component was represented by significant positive loading for BL, HG, PG, FLL and HLL (Table-3).

**Table-2:** Total variance explained by different component in adult hill cattle of Himachal Pradesh.

Component	Initial eigen values			Extraction sum of square loadings		
	
	Eigenvalue	Percentage of variance	Cum percent	Total	Percentage of variance	Cum percent
1	5.891	34.66	34.66	3.489	20.52	20.52
2	1.757	10.34	45.00	2.341	13.77	34.29
3	1.295	7.62	52.61	1.938	11.40	45.69
4	1.254	7.37	59.99	1.931	11.36	57.05
5	1.013	5.96	65.95	1.512	8.90	65.95
6	0.940	5.53	71.48			
7	0.848	4.99	76.46			
8	0.653	3.84	80.31			
9	0.630	3.70	84.01			
10	0.595	3.50	87.51			
11	0.477	2.80	90.32			
12	0.410	2.41	92.73			
13	0.364	2.14	94.88			
14	0.332	1.96	96.83			
15	0.213	1.26	98.09			
16	0.173	1.02	99.11			
17	0.151	0.89	100.00			

**Table-3:** Varimax rotated loading matrix and communality estimate for different biometric traits of hill cattle.

Traits	Principal components					Communality

	1	2	3	4	5	
BL	0.60	0.36	0.21	0.25	0.06	0.60
HG	0.73	0.13	0.35	−0.06	0.19	0.72
PG	0.73	0.19	0.29	−0.10	0.16	0.70
FLL	0.75	−0.03	−0.15	0.27	−0.15	0.68
HLL	0.70	0.06	−0.09	0.25	−0.08	0.58
FL	0.53	0.28	0.39	0.14	−0.26	0.59
FOL	0.07	0.09	0.07	0.84	0.02	0.73
FOW	0.02	0.24	0.25	0.74	−0.13	0.73
HH	0.56	0.44	0.44	0.30	0.07	0.80
HL	0.17	0.08	0.27	0.44	0.33	0.41
HHD	0.49	0.27	0.17	−0.10	0.55	0.66
PPD	−0.02	0.01	0.05	−0.01	0.67	0.45
TL	0.12	0.92	0.12	0.13	−0.02	0.89
TUS	0.22	0.88	0.11	0.17	−0.02	0.88
HL	0.19	0.24	0.65	0.24	−0.23	0.66
HC	0.06	0.05	0.76	0.15	0.09	0.62
EL	0.07	0.29	0.29	−0.06	−0.06	0.49

BL=Body length, HG=Heart girth, PG=Paunch girth, FLL=Forelimb length, HLL=Hind limb length, FL=Face length, FOL=Forehead length, FOW=Forehead width, HH=Height at hump, HL=Hump length, HHD=Hook to hook distance, PPD=Pin to pin distance, TL=Tail length, TUS=Tail length up to switch, HL=Horn length, HC=Horn circumference, EL=Ear length

The first component describes the general body size of hill cattle. Various other studies on beef cattle [12], in black Japanese cattle [13] and in Assam hill cattle [10] reported that the first principal component was measure of overall body size. The proportion of total variance explained by the first component was more in present investigation compared to earlier study [10] in Assam hill cattle (21.93%) and in Gojri buffalo [5] (31.4%), but lowered than those reported [14] in White Fulani cattle. The second factor which account for 10.3% of variation revealed significantly high loading for tail characteristics. The third factor explained 7.6% of variation and had higher loading for horn characteristics. The fourth component accounted for 7.4% of the variation and represented by high loading for facial characteristics. The fifth factor which accounted for 5.9% of variation was represented by higher loading for distance between hook bones and distance between pin bones, which tends to describe rear body of animal. The communality estimate ranged from 0.41 (HL) to 0.88 (TL). Communality estimate for few traits were lower than earlier reports [14] in White Fulani cattle (0.79-0.93). Lower communality estimate for HL, PPD and EL indicates that probably they did not explain body confirmation in hill cattle. Inter-factor correlation coefficient ranged from −0.52 to 0.6, with the first factor showed a positive correlation with other.

## Conclusion

The first factor contributes effectively to explain general body confirmation of hill cattle of Himachal Pradesh. The positive and significant correlation among different biometrical traits also makes them amenable for analysis. The five factors extracted from the present investigation could be used to select animals based on a group of variables rather than isolated traits. The similar selection strategy has been recommended by earlier findings [14,15]. The result of present investigation suggested that indigenous hill cattle of Himachal Pradesh are small and compact size cattle with medium hump, horizontally placed short ears and long tail. The study also revealed that factors extracted from the present investigation could be used in breeding programs with sufficient reduction in the number of biometric traits to be recorded to explain the body confirmation.

## Authors’ Contributions

SK and YP designed the plan and organized the study. DV carried out the study and collected the data, VS coordinated the data- analysis and drafted and revised the manuscript, All authors read and approved the final manuscript.
